# Impact of planning organ at risk volume margins and matching method on late gastrointestinal toxicity in moderately hypofractionated IMRT for locally advanced pancreatic ductal adenocarcinoma

**DOI:** 10.1186/s13014-023-02288-3

**Published:** 2023-06-19

**Authors:** Ayaka Ogawa, Michio Yoshimura, Mitsuhiro Nakamura, Takanori Adachi, Takahiro Iwai, Ryo Ashida, Takashi Mizowaki

**Affiliations:** 1 Department of Radiation Oncology and Image-Applied Therapy, Graduate School of Medicine, Kyoto University, 54 Kawahara-cho, Shogoin, Sakyo-ku, Kyoto, 606-8507 Japan; 2 Department of Advanced Medical Physics, Graduate School of Medicine, Kyoto University, 53 Kawahara-cho, Shogoin, Sakyo-ku, Kyoto, 606-8507 Japan; 3 Department of Radiation Oncology, Kobe City Medical Center General Hospital, 2-1-1, Minatojima Minamimachi, Chuo-ku, Kobe, 650-0047 Japan

**Keywords:** GI bleeding, Dosimetric analysis, Pancreatic cancer, PRV margin, Fiducial marker

## Abstract

**Background:**

This study examined the differences in late gastrointestinal (GI) toxicities in moderately hypofractionated intensity-modulated radiation therapy (IMRT) for locally advanced pancreatic ductal adenocarcinoma (LA-PDAC) by changing the planning organs at risk volume (PRV) margin and the target matching method and assessed the causes of adverse events.

**Methods:**

We examined 37 patients with LA-PDAC who underwent moderately hypofractionated IMRT between 2016 and 2020 at our institution; 23 patients were treated with wide PRV margins and soft tissue matching (Protocol A) and 14 with narrow PRV margins and fiducial marker matching (Protocol B). The GI toxicities, local control (LC) rate, and overall survival (OS) were assessed for each protocol. The initially planned and daily doses to the gross tumor volume (GTV), stomach, and duodenum, reproduced from cone-beam computed tomography, were evaluated.

**Results:**

The late GI toxicity rate of grades 3–4 was higher in Protocol B (42.9%) than in Protocol A (4.3%). Although the 2-year LC rates were significantly higher in Protocol B (90.0%) than in Protocol A (33.3%), no significant difference was observed in OS rates. In the initial plan, no deviations were found for the stomach and duodenum from the dose constraints in either protocol. In contrast, daily dose evaluation for the stomach to duodenal bulb revealed that the frequency of deviation of V_3 Gy_ per session was 44.8% in Protocol B, which was significantly higher than the 24.3% in Protocol A.

**Conclusions:**

Reducing PRV margins with fiducial marker matching increased GI toxicities in exchange for improved LC. Daily dose analysis indicated the trade-off between the GTV dose coverage and the irradiated doses to the GI. This study showed that even with strict matching methods, the PRV margin could not be reduced safely because of GI inter-fractional error, which is expected to be resolved with online adaptive radiotherapy.

**Supplementary Information:**

The online version contains supplementary material available at 10.1186/s13014-023-02288-3.

## Background

The prognosis of pancreatic ductal adenocarcinoma (PDAC) is poor, with a 5-year survival rate of < 10%. Due to vascular involvement at the initial diagnosis, many patients have locally advanced diseases, making them ineligible for surgery [1]. Although several novel chemotherapy regimens have slightly improved the prognosis of PDAC [2], only a few cases of locally advanced pancreatic ductal adenocarcinoma (LA-PDAC) can be completely cured by definitive chemoradiotherapy or conversion surgery. Therefore, additional multidisciplinary therapy, including radiotherapy, is required.

As the results of radiation therapy for LA-PDAC have been unsatisfactory [3], there is an urgent need to increase the radiation intensity. Recent innovations in high-precision radiotherapy, including intensity-modulated radiation therapy (IMRT) and stereotactic body radiation therapy (SBRT), have resulted in local dose escalation and improved treatment outcomes [4, 5]. However, increasing the target dose while maintaining a low dose for organs at risk (OARs) is still difficult because the pancreas is surrounded by radiosensitive organs, including the stomach and duodenum, which are deformed by daily variation or peristalsis. Furthermore, experienced institutions have reported grade 4 or greater adverse events in patients treated in SBRT dose-escalation trials [6]. Therefore, safe and effective radiation treatment for PDAC requires the optimal prescribed dose and a balance of various technical factors, including appropriate planning organs at risk volume (PRV) margins, respiratory management, and target matching methods. Our institution initiated moderately hypofractionated IMRT for LA-PDAC in 2009. First, we conducted a phase I dose-escalation study to determine the maximum tolerated dose with a full dose of gemcitabine (UMIN000004589). Finally, we decided on 48 Gy in 15 fractions as a prescription dose for LA-PDAC. We continued this dose fractionation for several years and achieved favorable outcomes with acceptable toxicity [7]. Additionally, the following two factors of the IMRT protocol were changed to further improve treatment outcomes: the PRV margin and the matching method. After revising the protocols, a series of late gastrointestinal (GI) adverse events made it necessary to revert the protocol. Unexpected daily dose deviations of GI by computed tomography (CT) on rail or magnetic resonance (MR)-guided adaptive radiation therapy for abdominal tumours have been shown [8, 9], and the potential risk of image guidance with implanted markers for margin reduction around the clinical target volume (CTV) has been reported for prostate cancer [10]. However, no reports have shown that the alteration of CTV/PRV margins and matching method for IMRT protocol affected the toxicity in PDAC. Therefore, this study aimed to examine the differences in the late GI toxicities in IMRT for LA-PDAC by changing the PRV margin and matching method, and to assess the causes of adverse events.

## Methods

### Patients

Ethical approval was obtained before the study (approval number: R1048). Patients with LA-PDAC who underwent definitive exhalation breath-hold IMRT between February 2016 and July 2020 at our institution were retrospectively reviewed. Additionally, all patients were pathologically diagnosed with PDAC. Patients with recurrent PDAC who had already undergone duodenectomy were excluded. Patient, tumor, and treatment characteristics were obtained from medical records. After RT, patients were followed up once every 1–2 months, and all toxicities were scored using the Common Terminology Criteria for Adverse Events, version 5.0. Late GI toxicities were defined as occurring 3 months or later from the starting date of radiotherapy, and if multiple symptoms were present, the date of the first event after 3 months was recorded as the interval.

### Radiotherapy

**Fig. 1:**
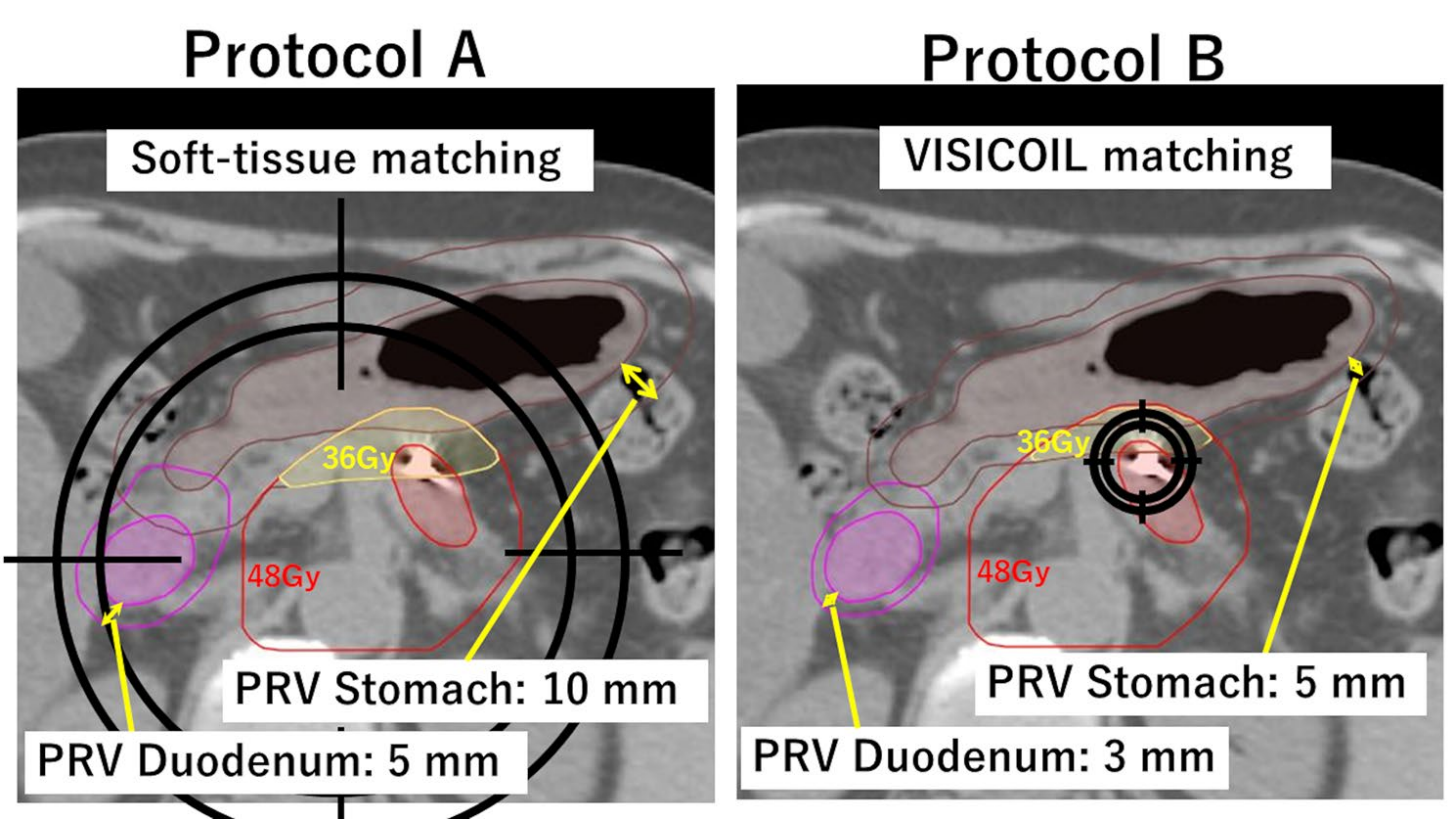
An example showing the differences between Protocols A and B The red shading represents the GTV, and the red line represents the PTV. The PRV (planning organs at risk volume) margin was 10 mm for the stomach and 5 mm for the duodenum in Protocol A (**a**) and 5 mm for the stomach and 3 mm for the duodenum in Protocol B (**b**). Alignment was determined using (**a**) soft tissue and (**b**) VISICOIL. In both protocols, 48 Gy was prescribed for D_95%_ of PTV minus PRV, whereas D_98%_ of PTV was 36 Gy or more. GTV, gross tumour volume; PRV, planning organs at risk volume; CTV, clinical target volume; PTV, planning target volume

Patients were treated using two radiation protocols, which differed in PRV margins and matching method (Fig. 1). The details of this process are described below. Protocol A was applied to patients irradiated from February to July 2016 and subsequently changed to Protocol B; thereafter, it was returned to Protocol A in February 2018. For all patients in Protocol B, one VISICOIL (RadioMed Corporation, Maryland, USA) was inserted trans-endoscopically inside or near the tumor under endoscopic ultrasound before planning the CT. Notably, one patient during the period of Protocol B was treated with Protocol A because VISICOIL had migrated before irradiation.

Non-contrast and transvenous contrast-enhanced CTs with 2 mm slices were acquired under the condition of end exhalation breath-hold for planning using an individualized vacuum pillow (Body Fix; Elekta, Stockholm, Sweden) and a real-time position management system (RPM; Varian Medical Systems). The gross tumor volume (GTV) included the primary tumor and metastatic lymph nodes. The CTV was defined as the GTV plus a 5 mm margin and retropancreatic regions between the celiac axis and superior mesenteric artery. Additionally, a planning target volume (PTV) margin of 5 mm was isotropically added to the CTV. The surrounding OARs, including the stomach, duodenum, small bowel, large bowel, liver, kidneys, and spinal cord, were also contoured. Different PRV margins were applied depending on the protocols. For Protocol A, 5 and 10 mm were added to the duodenum and stomach, respectively, based on a study conducted at our institution, which investigated the proper PRV margins [11]; for Protocol B, 3 and 5 mm were added, respectively (Fig. 1). At least two board-certified radiation oncologists defined these structures.

IMRT planning was performed as in previous reports of our institution [7, 12]. Overall, the prescription dose was 48 Gy in 15 fractions, covering 95% of the target (D_95%_) to a volume that subtracted PRVs from the PTV, and PTV D_98%_ ≥ 36 Gy was preferable. A 5% reduction in the prescribed dose or D_50%_ prescription was ensured when the OAR dose constraints could not be achieved. The dose constraints of the OARs were also the same as in our previous reports; for example, for the stomach and duodenum, V_45 Gy_ < 1 cm^3^, V_42 Gy_ < 5 cm^3^, and V_39 Gy_ < 25 cm^3^ were to be met.

On the treatment day, cone-beam CT (CBCT) was performed immediately before beam delivery under the condition of end exhalation breath-hold using the RPM system to confirm the positioning of the target. In Protocol A, the patient setup was based on soft tissues, including the pancreas and large blood vessels. Specifically, because tumor and prophylactic lymph node areas frequently had different deviations, we selected an acceptable middle ground for both and then confirmed the presence of the GTV within the PTV. Subsequently, we verified the position of the intestine in relation to the PTV and made adjustments if necessary. In contrast, in Protocol B, the patient setup was performed using VISICOIL (Fig. 1). Furthermore, VISICOIL was monitored to observe if it was within 2 mm during beam delivery, and when it exceeded that range, CBCT was performed again [13]. For the patients in Protocol B, backup plans were generated using the same procedure as Protocol A in case VISICOIL could not be recognized during irradiation. Finally, the treatment beam was delivered using TrueBeam STx (Varian Medical Systems) under the condition of end exhalation breath-holding.

### Chemotherapy

The main induction chemotherapy regimen was a weekly intravenous administration of 1000 mg/m^2^ gemcitabine. The regimen was decided based on the performance status by the tumor board of our institution or the referring physician, and at least one course was administered to all patients. During IMRT, weekly intravenous gemcitabine (1000 mg/m^2^) was administered, or S-1 (80 mg/m^2^/day) was administered on weekdays if there were allergies to gemcitabine or other problems. Additional treatment after radiotherapy mainly comprised gemcitabine monotherapy until tumor progression or patient refusal, and the regimen after recurrence was determined by the tumor board of our institution.

### Dose distribution analysis

The dose distribution was secondarily analyzed to determine whether late adverse events depended on protocol changes. The initial plan’s GTV and gastric and duodenal doses were compared in Protocols A and B. The backup plan for patients in Protocol B, which was made during the treatment planning using the same procedure as Protocol A, called Plan A’ was also compared to the initial plan in Protocol A to evaluate differences in patient anatomy and optimization bias.

For the patients in Protocol B, the daily structures of GTV and StoDuo defined from the stomach to the duodenal bulb, which is a favorable site for ulceration even in the absence of RT [14], were retrospectively contoured on daily CBCT [all 210 sessions (14 patients × 15 fractions)]. Subsequently, these structures were superimposed on the initial CT with VISICOIL match, and the daily dose to GTV and StoDuo was evaluated with the initial plan. Furthermore, to simulate Protocol A treatment in 14 patients treated with Protocol B, a board-certified radiation oncologist retrospectively conducted soft tissue matching, and the daily dose of the GTV and StoDuo were examined with initial Plan A’ (referred to as Protocol A’). Notably, deformable registration was not used during these processes.

### Statistical analysis

The characteristics of patients treated with the protocols were compared using Fisher’s and Wilcoxon rank-sum exact tests. Overall survival (OS) was defined as the period from the starting date of chemotherapy to the date of death from any cause and was censored on the last follow-up day for living patients. Additionally, local control (LC) was defined as the period from the date of chemotherapy initiation to recurrence confirmed using CT, fluorodeoxyglucose positron emission tomography, or MR imaging. The OS and LC rates and the cumulative incidence rates of late GI toxicities were estimated using the Kaplan–Meier method. The log-rank test was performed for OS, LC, and GI toxicity rate comparisons.

Dose-volumetric indices of the initial plans in the stomach, duodenum, and GTV were analyzed statistically between Protocols A and B, as well as A and A’ using the Bonferroni correction with the Kruskal–Wallis test. Additionally, the Wilcoxon rank-sum test was used for Dose-volumetric indices of the daily dose of Stoduo and GTV between Protocols B and A’. All statistical tests were two-sided, and the significance level was 5%. All statistical analyses were performed using EZR (Saitama Medical Center, Jichi Medical University, Japan), a modified version of the R commander designed to add statistical functions commonly used in biostatistics [15].

## Results

### Patients’ characteristics

Overall, 37 patients were analyzed: 23 and 14 patients were treated with Protocols A and B, respectively. Additionally, two and four patients in Protocols A and B with suspected para-aortic lymph node metastasis or resolved liver metastasis were included, respectively. Patient characteristics and treatment details are presented in Table 1.

**Table 1:** Patient characteristics of tumors and treatment

Characteristic	Protocol A	Protocol B	*p*-value
Number of patients	23	14	
Age (median, range [years])	70 (53–83)	72.5 (48–86)	0.61
Gender (male/female)	15/8	6/8	0.31
PS (0/1/2)	13/9/1	10/4/0	
Tumor location (head/body-tail)	9/14	10/4	0.091
Pretreatment CA19-9[U/ml]	298 (8.7–2368)	280 (6.1–2715)	0.96
GTV (median, range[cm^3^])	31.46 (10.8–89.6)	21.65 (2.4–86.5)	0.11
PTV (median, range[cm^3^])	212.6 (146.2–483.4)	193 (129.4–452.3)	0.38
Overlap^*^ (median, range[cm^3^])	43.8 (12.4–105.2)	33.8 (9.7–85.4)	0.067
Induction Chemo-regimen (GEM/S1/ mFOLFIRINOX/GnP)	12/2/3/6	7/2/0/5	
Concurrent Chemo-regimen (GEM/S1)	19/4	12/2	1
PPI prescription^**^	21	14	0.51
NSAIDs prescription ^***^	3	2	1

Abbreviations: PS, performance status; GTV, gross tumor volume; PTV, planning target volume; GEM, gemcitabine; mFOLFIRINOX, modified FOLFIRINOX; GnP, gemcitabine plus nab-paclitaxel; PPI, proton pump inhibitor; NSAIDs, non-steroidal anti-inflammatory drugs

Note: ^*^Overlap indicates overlapping areas of PTV and PRV

^**^PPI prescription was defined at least up to 3 months from RT

^***^NSAIDs prescription was defined at least > 180 mg/day for more than one week

### Treatment outcome

**Fig. 2:**
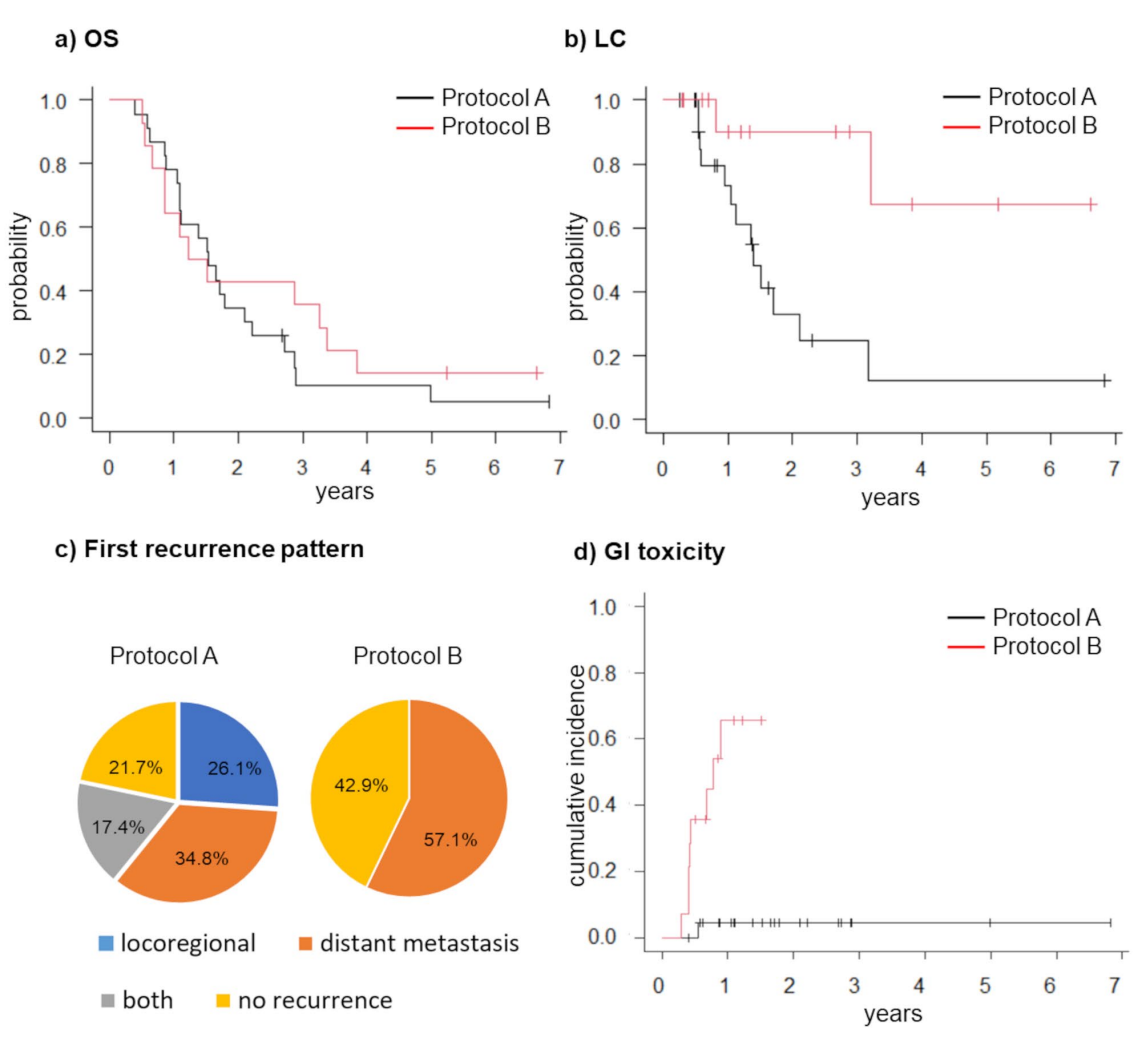
Treatment outcome of each protocol Kaplan–Meier estimates of (**a**) overall survival and (**b**) local control according to Protocols A (*n* = 23) and B (*n* = 14). Pie chart showing (**c**) the first recurrence pattern per protocol. Kaplan–Meier estimates of (**d**) cumulative incidence of GI toxicity GI, gastrointestinal

The median follow-up period was 18.5 months for all patients. The median survival time and 2-year OS rate were 18.4 months and 34.8% (95% confidence interval (CI) = 16.6–53.7%) in Protocol A, and 16.5 months and 42.9% (95% CI = 17.7–66.0%) in Protocol B, respectively, and the OS was not statistically different (*p* = 0.492) (Fig. 2a). During the analysis, 33 patients died, 4 (2 and 2 in Protocols A and B, respectively) remained alive, and 3 survived more than 5 years. The 2-year LC rates in Protocol A and B groups were 33.0% (95% CI = 11.7–56.4%) and 90.0% (95% CI = 47.3–98.5%), respectively. The LC of Protocol B was significantly better than that of Protocol A (*p* < 0.05) (Fig. 2b). Regarding the recurrence pattern, the first recurrence occurred in the locoregional area, distant organs, and both locoregional and distant metastases in six (26.1%), eight (34.8%), and four (17.4%) patients in Protocol A, respectively. In contrast, in Protocol B, no patient had a locoregional relapse as the first relapse, and eight (57.1%) patients had distant metastasis as the first relapse (Fig. 2c). One of the locoregional recurrences was found to be both local and regional lymph node recurrence, and the rest were local recurrences. More details of the recurrence are presented in Supplement Table 1.

### Gastrointestinal toxicities

Grade 2 GI toxicities were not observed in Protocol (A) In contrast, two (14.3%) patients were observed in Protocol (B) The incidence of grades 3–4 GI toxicities was 4.3% (one patient) and 42.9% (six patients) in Protocols A and B, respectively. None of the patients had grade 5 adverse events in either protocol. The median duration of adverse events from the start of irradiation was 5.5 months (4.9–10.9 months) (Table 2). Five and two cases of bleeding were due to upper GI bleeding caused by ulceration or gastritis and intra-abdominal bleeding due to rupture of the pseudoaneurysm, respectively. All ulcers or gastritis were located between the gastric angle and the duodenal bulb (Table 2). A graph of the cumulative incidence of adverse events is shown in Fig. 2d.

**Table 2:** Summary of late gastrointestinal toxicity for all cases

Protocol	Age (yr)	Primary Site	TNM (UICC 7th)	GTV (cm^3^)	PTV (cm^3^)	Overlap (cm^3^)	Late toxicity	Grade (CTCAEv.5)	Form and Location	Interval^*^ (months)	OS^**^ (months)
B	48	Head	T4N0M0	31.3	178.7	43.4	Hemorrhage	Grade 4	Duodenal bulb ulcer	4.9	39.8
B	61	Head	T4N0M0	47.9	254.7	33.3	Ulcer	Grade 2	Stomach vestibule ulcer	10.9	41.1
B	69	Head	T4N1M1	53.2	452.3	366.9	Hemorrhage	Grade 3	Stomach vestibule gastritis	3.5	6.6
B	64	Head	T4N0M0	15.2	165.8	155.7	Hemorrhage +ulcer	Grade 4 Grade 2	GDA pseudoaneurysm Duodenal bulb ulcer	5.1	63.7
B	59	Head	T4N1M0	25.6	204.5	176.2	Hemorrhage	Grade 3	Stomach vestibule gastritis	5.3	10.3
B	66	Body	T3N0M1	4.0	129.4	119.7	Hemorrhage	Grade 3	Stomach vestibule gastritis	9.5	80.8
B	82	Head	T3N0M1	2.4	193.5	162.7	Ulcer	Grade 2	Stomach angle ulcer	8.2	46.8
B	56	Head	T4N1M1	21.9	197	156.9	Hemorrhage	Grade 4	Brunch of SMA pseudoaneurysm	5.5	35.0
A	73	Body	T3N1M1	74.6	483.4	105.2	Hemorrhage	Grade 3	Duodenal bulb ulcer	6.7	18.7

*Abbreviation*s: GTV, gross tumor volume; PTV, planning target volume; GDA, gastro duodenal artery; OS, overall survival; SMA, superior mesenteric artery

^*^ Calculated from start of radiotherapy, the first event date after 3 months was used

^**^ Calculated from start of chemotherapy

### Dose distribution analysis

**Fig. 3:**
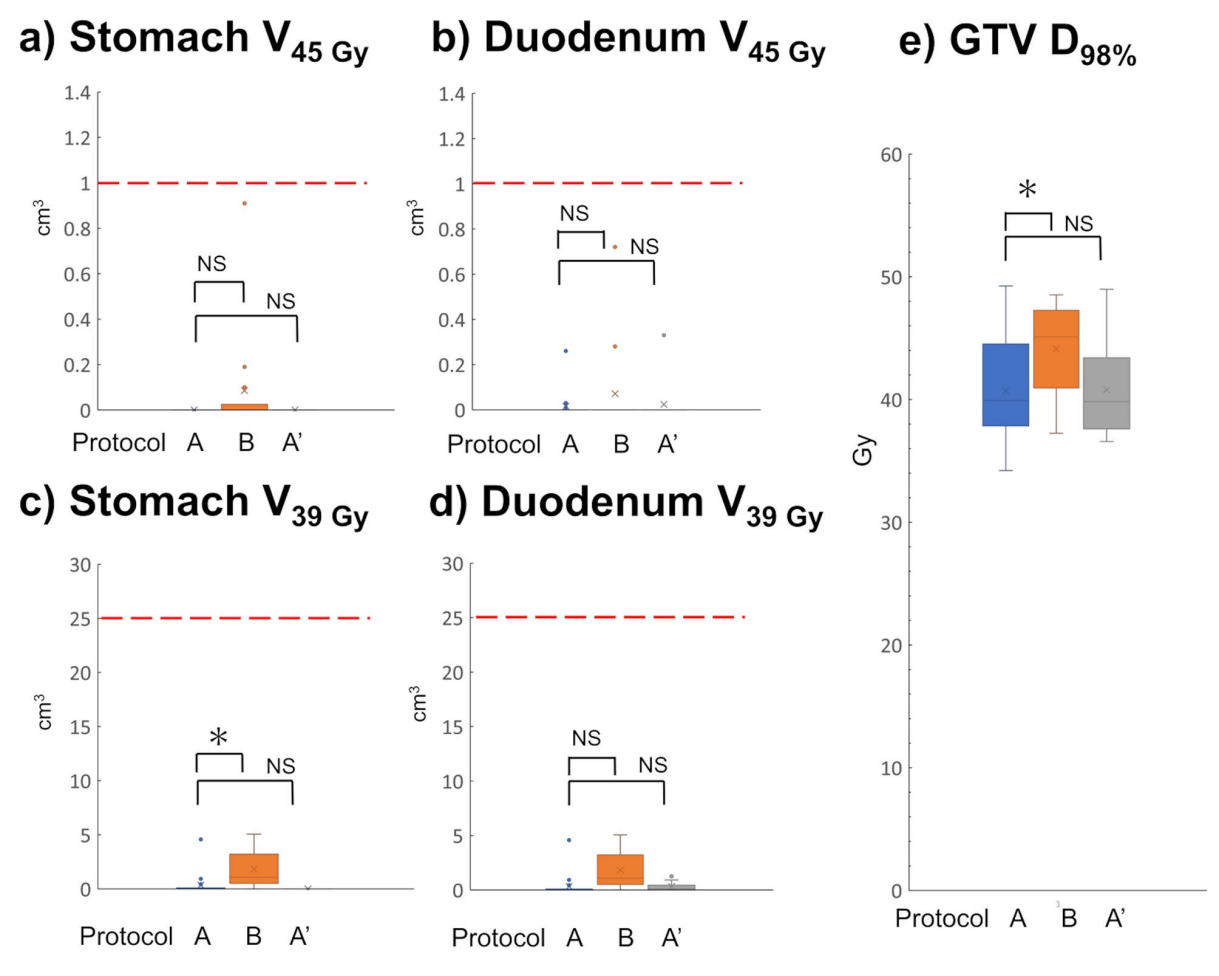
Box-and-whisker plot of dose indices in the initial plan per protocol Box-and-whisker plot of (**a**) V_45 Gy_ of the stomach (**b**) V_45 Gy_ of the duodenum (**c**) V_39 Gy_ of the stomach (**b**) V_39 Gy_ of the duodenum (**e**) GTV D_98%_ of the initial plan per Protocols A, B, and A’. Dots indicate abnormal data points. The dashed red line indicates the dose constraints of the stomach and duodenum at our institution. Protocols A and B, and A and A’, are compared; those with significant differences are marked with an asterisk, and those without significant differences are marked with NS. GTV, gross tumour volume

Figure 3 shows V_45 Gy_ and V_39 Gy_ of the stomach and duodenum and GTV D_98%_ for each protocol in the initial plan. Only V_39 Gy_ of the stomach in Protocol B (median: 1.1 cm^3^ [range, 0–5.1 cm^3^]) was statistically significantly greater than that in Protocol A (median: 0 cm^3^ [range, 0–4.6 cm^3^]) (*p* < 0.05); conversely, all values were sufficiently lower than the dose-volume constraints. Whereas GTV D_98%_ was higher in Protocol B than in Protocol A (*p* < 0.05). No significant differences were found for all parameters in the comparison between Protocols A and A’.

**Fig. 4:**
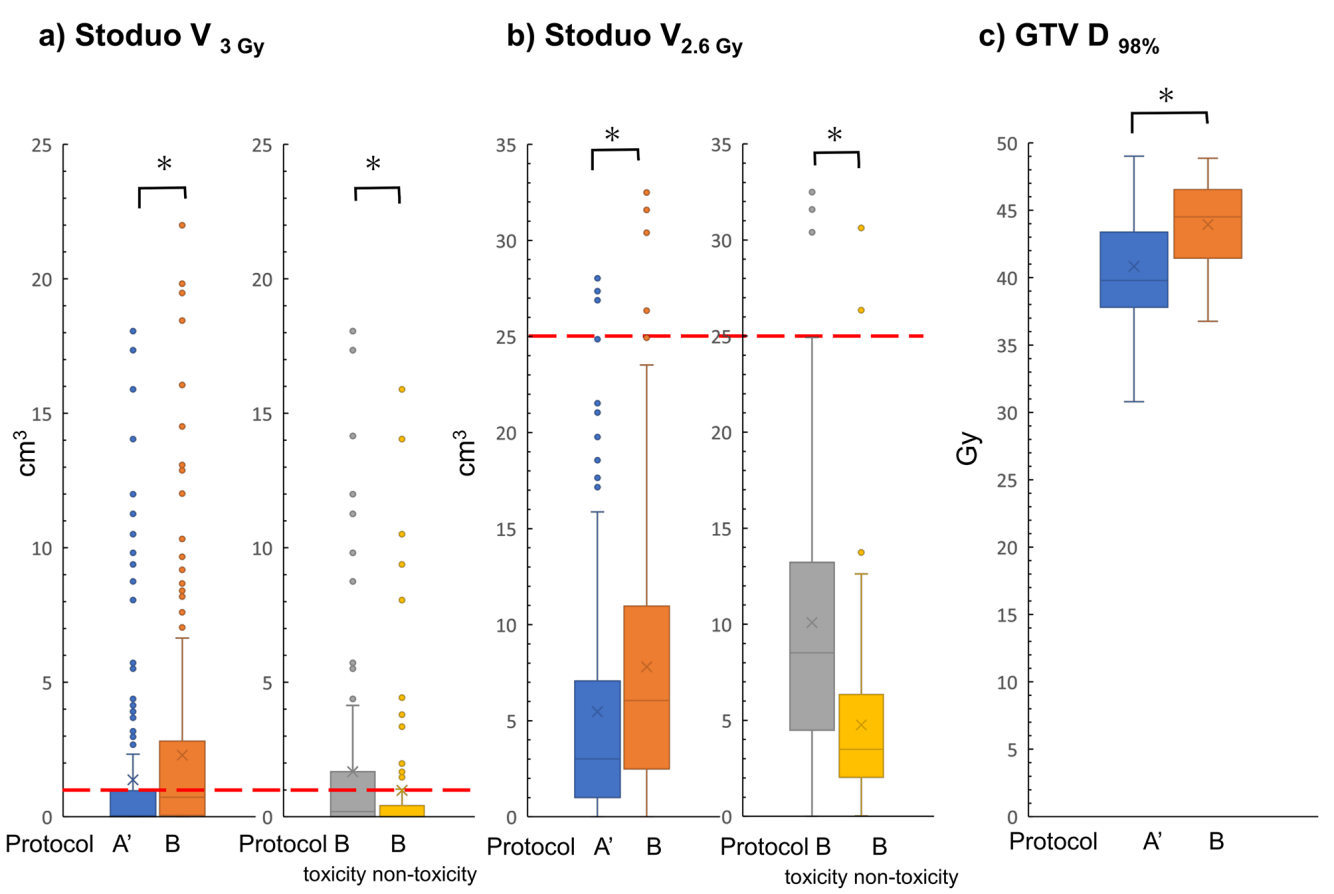
Box-and-whisker plot of daily dose indices to StoDuo and GTV per protocol Box-and-whisker plot of (**a**) daily V_3 Gy_ of Stoduo (equivalent to V_45 Gy_ of 15 fractions) (**b**) daily V_2.6 Gy_ of Stoduo (equivalent to V_39 Gy_ of 15 fractions) for Protocols A’ and B, in addition to the subgroup of toxicity patients group and non-toxicity patients group in Protocol B. (**c**) Box-and-whisker plot of daily GTV D_98%_ per Protocols A’ and B. Abnormal data points are indicated by dots. The dashed red lines indicate the dose constraints at our institution. Protocols A’ and B were compared, and significant differences are marked with an asterisk. GTV, gross tumour volume

The daily doses of StoDuo in Protocols B and A’ (reproduction of Protocol A in B patients) are shown in Fig. 4. In Protocol B, 94 of the 210 fractions (44.8%) deviated from the V_3 Gy_ < 1 cm^3^ constraint per session, which is a surrogate for the V_45 Gy_ < 1 cm^3^ in 15 fractions. In contrast, 51 of the 210 fractions (24.3%) deviated from the constraint in Protocol A’. The Wilcoxon rank-sum test showed that V_3 Gy_ of Protocol B was significant higher than that of Protocol A (*p* < 0.05) (Fig. 4a). A similar trend was observed for V_2.6 Gy_ per session (*p* < 0.05), which is a surrogate for V_39 Gy_ in 15 fractions (Fig. 4b). Furthermore, the median dose of V_3 Gy_ in the patient group with adverse events was 1.4 cm^3^ (range 0–22.0 cm^3^), whereas that in the group without adverse events was 0.18 cm^3^ (range 0–18.5 cm^3^), showing a statistical difference between the group with and without adverse events in Protocol B (*p* < 0.05). Regarding the daily target dose, the median GTV D_98%_ was 45.5 Gy (range 36.8–48.9 Gy) and 39.8 Gy (range 30.8–49.0 Gy) for Protocols B and A’, respectively. The GTV dose in Protocol B was significantly higher than that in Protocol A’ (*p* < 0.05).

## Discussion

This study aimed to examine the late GI toxicities in patients with LA-PDAC treated with moderately hypofractionated IMRT with two different treatment policies and to evaluate the causes of adverse events. We have been conducting exhalation breath-hold IMRT for LA-PDAC since 2009 [7, 12] and changed our treatment strategy from Protocol A to Protocol B, which used narrower PRV margins and VISICOIL matching. We expected to achieve better outcomes by changing the protocol because reducing the PRV margin increases the volume of PTV irradiated at 48 Gy instead of 36 Gy. However, the incidence of grade 3 or higher GI toxicity was 6 of the 14 (42.9%) cases in Protocol B, which was higher than that in Protocol A (4.3%) and several previous reports [7, 12, 16, 17]. The rate of pancreatic head cancer was higher in Protocol B than in Protocol A, although this difference was not statistically significant (Table 1). A systematic review found that the incidence of grade 3 or higher late GI toxicity was significantly reduced from 10.6 to 5.0% with IMRT compared to three-dimensional (3D) conformal radiotherapy [17], and the trend was consistent with a previous report from our institution [7]. Although many dose-volume indices for the risk of late GI toxicity have been examined [16, 18, 19], they cannot be compared because they differ in fractionation, concomitant chemotherapy, irradiation techniques, and irradiation areas. For moderately hypofractionated IMRT, Cattaneo et al. reported that they delivered 44.25 Gy in 15 fractions in 61 patients with LA-PDAC. Some patients received 48–58 Gy as a simultaneous integrated boost under free breathing. The incidence of anatomical grade 3 toxicity, including ulcer or duodenitis, was 8.2%. They concluded that the high-dose region to the duodenum was strongly correlated with anatomical GI toxicity and that the best cut-off values of V_40 Gy_ and V_45 Gy_ were 16% (converting to a volume of approximately 11.7 cm^3^) and 2.6% (approximately 3.5 cm^3^), respectively [19]. Here, the dose-volume indices were reasonable, and no deviations were observed in the initial plans of Protocol B (Fig. 3a–d). Previous studies evaluated the risk of adverse events using only the initially planned dose-volume indices. However, it was reported that inter- and intra-fractional movements of OARs had large displacements (> 10 mm) for upper GI OARs [20]. Additionally, a study on CT on-rail imaging demonstrated that the actual OAR doses were significantly higher than the initially planned dose [8]. Therefore, we analyzed the daily dose of StoDuo using CBCT and found that there were 44.8% deviations in our V_3 Gy_ constraint per session, equivalent to V_45 Gy_ in 15 fractions. These results suggest that even with conformal irradiated regions in IMRT under the condition of respiratory motion control and strict matching of tumor location with VISICOIL, the PRV margin of Stoduo should not have been reduced because deformations of StoDuo were not linked to the tumor position on the day.

Meanwhile, Protocol B showed a better LC than Protocol A. Analysis of dose-volume indices of the initial plans demonstrated that Protocol B increased GTV D_98%_ more than Protocol A (*p* = 0.036) (Fig. 3e). Furthermore, in the daily dose with CBCT, Protocol B had a significantly increased daily GTV of V_98%_ compared with Protocol A’ (Fig. 4c). Krishnan et al. found that increasing the dose of BED_10_ to > 70 Gy (57.25 Gy in 25 fractions) only for patients who were more than 1 cm from the GTV to the intestinal tract showed a better OS [4]. In our study, the 48 Gy in 15 fractions is equivalent to 63.4 Gy at BED_10_ and is comparable to 70 Gy at BED_10_ when the irradiation period is shortened by 2 weeks to suppress tumor repopulation. Therefore, this improved LC rate in Protocol B might be due to the increased GTV dose. However, in this study, as with the chemoradiotherapy arm of the LAP07 trial, better LC did not improve OS because of potential metastases in many patients with LA-PDAC [3]. Conversely, LC may benefit patients without potential metastases or whose metastases can be controlled with recent aggressive chemotherapy. Indeed, several studies have shown favorable OS by increasing local irradiation doses [4, 5]. Although only approximately 1/3 of the patients in this study were treated by intensive induction chemotherapy, such as FOLFIRINOX or Gemcitabine plus nab-paclitaxel, favorable LC may lead to improved OS when we adopt intensive chemotherapy and appropriate patient selection based on biological characteristics. Additionally, enhancing LC is beneficial in preventing obstruction of the GI tract or bile ducts, cholangitis, and pain, which results in maintaining the quality of life and ensuring subsequent chemotherapy.

The balance between radiation intensity and adverse events is critical regarding the tolerability of subsequent chemotherapy in definitive chemoradiotherapy for pancreatic cancer. Reducing adverse events allows intensive chemotherapy to be continued. Protocol B had better LC (Fig. 2b), and the OS of patients with adverse events was relatively longer (Table 2); however, the incidence of adverse events was unacceptable (Fig. 2d). Because there were two variables (PRV margin and setup method), and their effects were not separated, determining which variable is responsible for this result is difficult, and interpreting these results requires caution. Even with high-precision radiotherapy, including IMRT or SBRT, dose escalation is limited by intestinal adverse events [18]. A recent phase I SBRT dose-escalation trial reported grades 4 and 5 late toxicities in patients with 45 Gy in 5 fractions [6]. Therefore, online adaptive irradiation may be a promising technology to overcome the issues of treatment intensity and adverse events. Our recent CBCT-based online adaptive study in silico revealed that without adaptive treatment, V_3 Gy_ < 1 cm^3^ of the stomach and duodenum per session was violated in 47 (28.5%) and 48 (29.1%) of 165 sessions, respectively. In contrast, no constraint violations were observed with adaptive treatment. The initial treatment plan, including prescription doses and margins, had planning conditions similar to those in Protocol B in this study [21]. Reyngold et al. delivered ablative dose escalation to 67.5 Gy in 15 fractions or 75 Gy in 25 fractions in some patients using the online adaptive method, demonstrating good OS [5, 22]. Other dose-escalation trials of stereotactic MR-guided adaptive RT reported that the GI toxicity was acceptable [23, 24]. Therefore, online adaptive therapy can adapt to a large inter-fractional motion and might be useful for safe dose escalation for PDAC.

As with any retrospective small-size analysis, this study had some limitations. First, the daily dose evaluation in this study was not a direct comparison between Protocols A and B but a comparison between Protocols A’ and B for the same patients using backup plans. A direct comparison would have been preferable; however, a comparison using the same cohort would have been appropriate to examine differences in position and PRV margin due to contouring errors described below. Second, we contoured the daily structures using CBCT images; therefore, there could be contour errors due to intestinal artifacts. However, the image quality of CBCT has improved recently [25, 26] and could be evaluated by confirming the 3D imaging of the stomach to the duodenal bulb. Third, intra-fractional errors because of peristalsis and respiratory management were not considered. Therefore, in the future, it will be possible to analyze images obtained during irradiation using online adaptive techniques. Finally, chemotherapy regimens varied, with 62.2% of induction chemotherapy using either gemcitabine or S-1 alone. The high rate of single-agent regimens may be due to the treatment era and the high proportion of elderly patients.

## Conclusions

By reducing PRV margins with strict matching methods, we observed a higher incidence of late GI toxicity in exchange for better LC. The protocol change ensured a higher daily GTV dose while increasing the daily high-dose area in the stomach to the duodenal bulb, which is a possible cause of adverse events. This study revealed that the PRV margin could not be safely reduced even with VISICOIL matching due to GI inter-fractional motion. Therefore, the dilemma between the target and GI doses is expected to be resolved by adapting the interactional GI motion with online adaptive therapy.

### Electronic supplementary material

Below is the link to the electronic supplementary material.

Supplementary Material 1

## Data Availability

Research data are stored in an institutional repository and will be shared upon request to the corresponding author.

## Abbreviations

GI: Gastrointestinal
IMRT: Intensity-modulated radiation therapy
LA-PDAC: Locally advanced pancreatic ductal adenocarcinoma
LC: Local control
OS: Overall survival
GTV: Gross tumor volume
SBRT: Stereotactic body radiation therapy
OARs: Organs at risk
PRV: Planning organs at risk volume
CBCT: Cone-beam CT
MR: Magnetic resonance
CI: Confidence interval
CTV: Clinical target volume
CT: Computed tomography
PTV: Planning target volume
RPM: Real-time position management.
