# Correction: Impact of planning organ at risk volume margins and matching method on late gastrointestinal toxicity in moderately hypofractionated IMRT for locally advanced pancreatic ductal adenocarcinoma

**DOI:** 10.1186/s13014-023-02321-5

**Published:** 2023-08-23

**Authors:** Ayaka Ogawa, Michio Yoshimura, Mitsuhiro Nakamura, Takanori Adachi, Takahiro Iwai, Ryo Ashida, Takashi Mizowaki

**Affiliations:** 1 Department of Radiation Oncology and Image-Applied Therapy, Graduate School of Medicine, Kyoto University, 54 Kawahara-cho, Shogoin, Sakyo-ku, Kyoto, 606-8507 Japan; 2 Department of Advanced Medical Physics, Graduate School of Medicine, Kyoto University, 53 Kawahara- cho, Shogoin, Sakyo-ku, Kyoto, 606-8507 Japan; 3 Department of Radiation Oncology, Kobe City Medical Center General Hospital, 2-1-1, Minatojima Minamimachi, Chuo-ku, Kobe, 650-0047 Japan

**Radiation Oncology (2023) 18:103**

10.1186/s13014-023-02288-3.

After publication of this article [1], the authors reported that in Table 2, second column, erroneously extra characters/digits were added: Age became aAge, 48 became 448, 61 became 661, etc.

The original article [1] has been corrected.
