# Supplementary material for: Impact of planning organ at risk volume margins and matching method on late gastrointestinal toxicity in moderately hypofractionated IMRT for locally advanced pancreatic ductal adenocarcinoma
Source: Radiat Oncol. 2023 Jun 19;18:103. doi: 10.1186/s13014-023-02288-3 (PMC10280835; doi:10.1186/s13014-023-02288-3)
Supplement: Supplementary file 1 — Supplementary Material 1 [file 13014_2023_2288_MOESM1_ESM.docx]

**Supplement table 1.** Details of first recurrence pattern.

| Number of patients | Protocol A | Protocol B |
| --- | --- | --- |
| Total | 23 | 14 |
| Locoregional* | 6(26.1%) | 0 |
| Distant metastasis | 8(34.8%) | 8(57.1%) |
| Both (locoregional and metastasis) * | 4(17.4%) | 0 |
| None | 5(21.7%) | 6(42.9%) |
| Details of distant metastasis** |  |  |
| Peritoneal dissemination | 7 | 3 |
| Lung metastasis | 4 | 3 |
| Distant lymph node metastasis | 2 | 3 |
| Liver metastasis | 1 | 2 |
| Ovarian metastasis | 1 | 0 |
| Bone metastasis | 0 | 1 |

*Only one of these patients had local recurrence and regional lymph node recurrence at the same time, and the other 9 patients had local recurrence.

**The number of patients were overlapping each other
